# Development of lipid nanoparticles and liposomes reference materials (II): cytotoxic profiles

**DOI:** 10.1038/s41598-022-23013-2

**Published:** 2022-10-27

**Authors:** Krishnapriya Syama, Zygmunt J. Jakubek, Sam Chen, Josh Zaifman, Yuen Yi C. Tam, Shan Zou

**Affiliations:** 1 Metrology Research Centre, National Research Council Canada, 100 Sussex Drive, Ottawa, ON K1A 0R6 Canada; 2 Integrated Nanotherapeutics, Burnaby, BC V5G 4X4 Canada

**Keywords:** Nanoscale materials, Drug development

## Abstract

Lipid based nanocarriers are one of the most effective drug delivery systems that is evident from the recent COVID-19 mRNA vaccines. The main objective of this study was to evaluate toxicity of six lipid based formulations with three surface charges—anionic, neutral or cationic, to establish certified reference materials (CRMs) for liposomes and siRNA loaded lipid nanoparticles (LNP-siRNA). Cytotoxicity was assessed by a proliferation assay in adherent and non-adherent cell lines. High concentration of three LNP-siRNAs did not affect viability of suspension cells and LNP-siRNAs were non-toxic to adherent cells at conventionally used concentration. Systematic evaluation using multiple vials and repeated test runs of three liposomes and three LNP-siRNA formulations showed no toxicity in HL60 and A549 cells up to 128 and 16 µg/mL, respectively. Extended treatment and low concentration of LNPs did not affect the viability of suspension cells and adherent cells at 96 h. Interestingly, 80% of A549 and HL60 cells in 3D conditions were viable when treated with cationic LNP-siRNA for 48 h. Taken together, anionic, cationic and neutral lipid formulations were non-toxic to cells and may be explored further in order to develop them as drug carriers.

## Introduction

The rapid development of COVID-19 mRNA vaccines utilizes the technology of nucleic acid therapeutics. It has been known for more than 50 years now that genetic diseases could be targeted by delivering functional copies of the dysfunctional gene. United States Food and Drug Administration (FDA) has recently approved nucleic acid therapeutics for fighting various genetic disorders^1^. This nucleic acid based therapeutic regulates the gene expression by introduction of nucleic acids to cells in contrast to conventional therapies which target mainly proteins. However, our immune system is tuned to eliminate or destroy vectors carrying genetic materials and the endogenous nucleases can easily degrade the newly introduced nucleic acid molecules^2^. Hence, the delivery of nucleic acids requires a biocompatible and efficient carrier^1^. Currently used drug carriers for nucleic acids are nanocarriers that remain stable in circulation and can efficiently deliver the nucleic acid payload to the intracellular target sites^1^. As defined by the ISO 17034:2016 international standard, a reference material is a “material, sufficiently homogeneous and stable with respect to one or more specified properties, which has been established to be fit for its intended use in a measurement process^3^”. Although in most of the formulation development papers, the size, toxicity and stability of formulations were reported, yet rarely a sufficient systematic study is provided to assess reliability of the measurements and statistical significance of observed variability, being it day-to-day, formulation-to-formulation, or aliquot-to-aliquot particle variation. Availability of an internationally recognized lipid nanoparticle reference material would promote and perhaps even enable high quality lab-to-lab comparable characterization of lipid nanoparticles, particularly in the present context, characterization of their size, size distribution, size stability and toxicity profile, similarly to what has previously been observed following development of polystyrene (e.g. NIST SRM1963a), gold (NIST SRM8011, 8012, and 8013 series), or silica (e.g. ERM FD304 or FD102) nanoparticle size reference materials. It should also be mentioned that availability of reference materials is particularly important for assigning and comparing values of method defined measurands, such as Z-avr hydrodynamic diameter^4^.

Lipid nanoparticle (LNP) carriers are one of the most advanced non-viral delivery systems and is currently being used for COVID-19 mRNA vaccines developed by Moderna (mRNA-1273) and Pfizer-BioNTech (BNT162b2). These two vaccines conferred a protection of more than 90% against COVID-19^5,6^, which has benefited the public health in fighting this pandemic tremendously^7^. Generally speaking LNPs contain ionizable lipids, phospholipids, cholesterol and polyethylene glycol conjugated lipids (PEG-lipids) and are taken up by cells via endocytosis or phagocytosis^8,9^. LNP formulations that contain siRNA (LNP-siRNA) were reported to silence gene expression responsible for tumor formation in vitro and in vivo. A group of cationic lipids were identified which can efficiently deliver siRNA in human lung cancer cell lines^10^. One of the main advantages of LNP systems is their modularity; small molecules can be made into lipophilic prodrugs for facile incorporation into LNP systems. In 2018, after years of research, FDA approved the first LNP-siRNA drug, Onpattro, developed by Alnylam Pharmaceuticals for treating transthyretin mediated amyloidosis^11^. LNPs are known to be biocompatible, biodegradable, less toxic and immunogenic compared to other drug carriers^12^.

Advances in LNP design stemmed from the pioneering work on lipids and their ability to form stable bilayer vesicles. These vesicles, termed liposomes, were the very first lipid-based systems used for drug delivery. Liposomes were first reported in 1965 and consist of a phospholipid bilayer encapsulating an aqueous compartment^13^. Owing to their similarity in morphology to cell membranes and ability to incorporate drug molecules, they have been a topic of scientific research for more than 50 years now. The composition of liposomes can be altered by the addition of lipids, cholesterol and polymers like polyethylene glycol (PEG) in order to increase their potency, stability and bio distribution and decrease the toxicity and immunogenicity. Liposomes are known to be the optimal and successful delivery vehicles for biomolecules^14,15^. The ability of liposomes as drug carrier depends on their physical and chemical properties such as their composition, size, surface charge and lipid organization^14,16^. Anti-tumor drugs such as doxorubicin (Dox), daunorubicin and epirubicin could be encapsulated in liposomes and were reported to be efficient with reduced cardiotoxicity compared with conventional anthracyclines^14^. Liposomal Dox and PEGylated liposomal Dox (PLD) showed low toxicity and better cardiac safety in patients with breast and ovarian cancer^17,18^.

Interaction of liposomes with the complex biological milieu depends on the surface properties of the liposomes. In particular, surface charge plays an important role. Liposomes are termed as anionic, cationic and neutral based on the overall surface charge. The nature and density of the charge can be modified by modulating the composition of lipids used to form the liposomes. A neutral surface charge can result in aggregation of liposomes and low physical stability. It may result in liposomes escape to the extracellular space as these liposomes do not interact well with cells^19,20^. However, positively or negatively charged liposomes were found to be more advantageous than neutral liposomes. The surface charge on the lipids prevents aggregation and increases the liposome-cell interaction. Of these two charged liposomes, positively charged liposomes were found to be more efficient drug carriers in vitro and in vivo^14^.

Negatively charged liposomes, which consist of anionic lipids, are reported to be less stable than neutral or positively charged liposomes. Furthermore, they are quickly absorbed by the reticulo-endothelial system and may also lead to toxic side effects^21^. Therefore, anionic liposomes are not extensively used as drug carriers^14^.

In the case of cationic liposomes, the electrostatic interaction between positively charged lipids and negatively charged nucleic acid molecules make them more feasible for gene delivery^9^. Neutral phospholipids such as dioleoyl phosphatidylethanolamine (DOPE) or dioleoyl phosphatidylcholine and positively charged lipids like DC-cholesterol HCl, (3β-[N-(N′, N′-dimethylaminoethane)-carbamyl] cholesterol hydrochloride), DOTAP (1, 2-dioleoyl-3-trimethylammonium-propane [chloride salt]), DOBAQ (N-[4-carboxybenzyl]-N,N-dimethyl-2, 3-bis(oleoyloxy) propan-1-aminium) are commonly used^14^.

One of the primary requirements of a delivery system is that they should be non-toxic to cells. Liposomes have been used successfully for a number of small molecule therapeutics^22^ while LNPs are the most advanced drug carriers in the clinic for nucleic acid delivery. The physicochemical characteristic of these drug carriers plays a major role in eliminating their toxicity^23^. Several studies have reported that physicochemical attributes such as size, composition, charge, surface area, agglomeration and dispensability can affect the toxicity of liposomes and LNPs^24,25^. The stability of the excipients is also an important prerequisite for drug formulations^26^.

Three dimensional culture models have gained much attention in the recent decades as they closely mimic the in vivo conditions and hence are more suitable than conventional two dimensional cultures^27^. It would be worthwhile to evaluate the toxicity of lipid nanoparticles in 3D culture, as they are an intermediate system between 2D culture and in vivo models.

The complex composition of liposomes and LNPs is crucial in determining their safety, stability and delivery efficiency when used for clinical applications. Biopharmaceutical companies develop individual in-house references to validate and maintain the manufacturability and quality of these formulations. When regulatory agencies receive these submissions of drug products for approval, the characteristics of each product become unique and details of the methodology and quality control results are usually not comparable. This has largely prolonged the regulatory processes. Certified reference materials (CRMs) are a measurement standard which can be utilized to confirm the physical characteristics and quality of a given product and measurement protocols. In general, CRM is utilized to provide confidence of measurements. Currently, there are no CRMs released from national metrology institutes available for liposomes or LNPs. As part of the Innovative Solutions Program, the National Research Council Canada is in collaboration with the Integrated Nanotherapeutics Inc. to develop CRMs of stable liposome and LNP formulations^28^. These candidate CRM formulations are aimed to be with narrow size distributions at nanoscale and sub-micron size (see Ref.^28^ for the homogeneity and stability characterization), in order to support the robust and required physical characterization in the development of drug product submissions, streamline the regulatory approval process and improve the manufacturability of drug delivery formulations. In this report, we outline the toxicity measurements of candidate CRMs of 3 different sizes with 3 different surface charges (neutral, positive or negative). These CRMs do not contain active drug and are designed to help create a reference standard for analytic measurements, which is available in the public domain and also support the knowledge dissemination and community engagement.

## Materials and methods

### Reagents

Dulbecco’s Modified Eagle’s Medium (DMEM), Iscove’s Modified Dulbecco's Medium (IMDM), fetal bovine serum (FBS), 1% penicillin–streptomycin, Dulbecco’s Phosphate Buffered Saline (DPBS) and 0.5% trypsin EDTA were purchased from Gibco (Thermo Fisher Scientific, Canada).

### Cell lines

HL60 (Promyeloblast, acute promyelocytic leukemia); NB4 (promyeloblast, acute promyelocytic leukemia with t (15;17) translocation marker), NIH3T3 (Mouse embryonic fibroblasts) and A549 (Lung adenocarcinoma) cells were purchased from American Type Culture Collection (ATCC, USA). NB4, A549 and NIH3T3 cells were cultured in DMEM containing FBS (10%), and 100 U/ml penicillin/streptomycin. HL60 cells were grown in IMDM supplemented with 20% FBS and 100 U/ml penicillin/streptomycin. All cell lines were cultured in a 5% CO_2_, 95% humidified incubator at 37 °C.

### Lipid nanoparticle-siRNA (LNP-siRNA) and liposomes (HC)

Six different formulations of LNP-siRNAs and liposomes were produced by Integrated Nanotherapeutics Inc. (INT, Vancouver, BC, Canada). LNP-siRNAs and liposomes (HCs) were prepared using their proprietary lipids and scaffold technology. LNP-siRNAs and HCs of 3 different sizes and 3 charges were stored at − 70 °C freezer and defrosted at room temperature for 40 min before any measurements. More details can be found in ref. 4, supporting information and below in Table 1.

**Table 1:** Naming of the liposome and lipid nanoparticle formulations.

Formulations	Short names	Lipid concentration (mg/mL)	Zeta potential (mV)	siRNA/lipid mass ratio
Anionic LNP-siRNA	ALNP	2	− 26.1	0.0419
Neutral LNP-siRNA	NLNP	3	2.4	0.0412
Cationic LNP-siRNA	CLNP	2	13.7	0.0341
Anionic Liposome	AHC	2	− 33.5	–
Neutral Liposome	NHC	2	− 1.9	–
Cationic Liposome	CHC	2	6.2	–

INT’s proprietary lipids and scaffold technology were applied in the formulations.

For the quantification of siRNA encapsulated in LNPs, standard siRNA and lipid materials corresponding to three LNP-siRNA formulations were also provided by INT.

### Cytotoxicity measurements

Cells were seeded in a 96-well microplate (HL60 at 25,000 cells/well, NB4 at 10,000 cells/well, A549 at 7000 cells/well, and NIH3T3 at 5000 cells/well). Each condition was replicated in 6 wells and 50 µL of cell suspension was dispensed in each well. For background measurement, 100 µL of respective media was added to 6 wells. Toxicity studies on 3D culture was carried out by seeding A549 and HL60 cells (1000 cells/well) in an agarose (1.5%) coated U-bottom 96-well plate. After 2–3 days, once the spheres were formed, cationic LNP-siRNA (0–128 µg/mL) was added and incubated for 48 h at 37 °C, 5% CO_2_. Viability in percentage is calculated following the below equation

$$Viability\left(\%\right)=\frac{O{D}_{sample, n}-O{D}_{blank}}{O{D}_{control}-O{D}_{blank}}\times 100\%$$

where *OD*_sample, n_, *OD*_blank_ and *OD*_control_ are the absorbance values of the cells treated with each formulation, the respective culture media and the cells without any treatment, respectively.

LNP-siRNAs and liposomes were diluted in 0.5X dPBS to final concentrations of 16, 64, 128, 256 and 512 µg/mL (unless otherwise stated) in specific media to a final volume of 50 µL and proper formulation was added to respective wells. Following incubation of the cells for 24, 48, 72 and 96 h, 10 µL of water soluble tetrazolium (WST)-8 reagent was added to each well. The plates were incubated for 4 h at 37 °C with 5% CO_2_ and the absorbance was measured at 450 nm using a plate reader (FLUOstar Omega, BMG LABTECH).

### Size distribution, size stability and zeta potential measurements

Size measurements were performed by dynamic light scattering (DLS) using a Zetasizer Nano ZS particle size analyzer (Malvern Instruments Ltd., Worcestershire, UK). Measurements were controlled and analyzed with Malvern Zetasizer Software ver. 7.11 unless otherwise stated. Formulations were diluted at room temperatures to 40 μg/mL in 1X dPBS except for NLNP-siRNA, which was 60 μg/mL. All measurements were performed at 25 °C using semi-micro polystyrene disposable cuvettes (VWR, USA). Following thermal equilibration in a sample compartment for minimum of 180 s, five repeat indications were acquired for each sample, with each indication being an average of eleven 10 s long runs. Z-average hydrodynamic diameter (Z-avr) and polydispersity (PdI) values were determined by the cumulants method with the Zetasizer Software parameters set at their default values. The five indications for each sample were averaged and the mean Z-avr and PdI values with corresponding standard deviations were reported. The cumulants analysis is a robust method applicable to monomodal and nearly monodisperse samples. With increasing particle aggregation, applicability of the cumulants analysis may be limited with Z-avr and PdI taking effective values not directly indicative of the size and size distribution of primary particles. In such cases, the measurements were further evaluated using the non-negative least squares (NNLS) distribution analysis.

Systematic studies on size distribution and stability at formulation storage temperature of − 70 and 4 °C, respectively, were reported in Ref.^4^, which followed the ISO17034:2016 standards. LNP-siRNA and liposome formulation stability tests at 37 °C are followed by measuring the size distributions after storage of formulations at 37 °C for 1 − 7 days.

For selected representative vials, electrophoretic mobility and zeta potential were measured with Zetasizer Nano ZS by mixed mode measurement phase analysis light scattering (M3-PALS). Details are included in ref. 4 and supporting information.

### Quantification of siRNA measurements

Standard siRNA at a stock concentration of 47.2 mg/mL was diluted to 5, 10, 15 and 20 µg/mL in a buffer (1% SDS in 0.5X dPBS) with respective lipid (0.2, 0.3, 0.4 and 0.5 mg/mL) and sucrose (50, 75, 100 and 125 mg, respectively). Absorbance was measured with a Nanodrop spectrophotometer (Nanodrop^TM^ One, Thermoscientific) and standard curves were plotted. Each LNP-siRNA was then diluted in 1% SDS-0.5X dPBS, absorbance was measured and the concentration of siRNA was calculated from the standard curve. The buffer containing corresponding lipid, sucrose and 1% SDS-0.5X dPBS served as the reference for each formulation of LNP-siRNA.

siRNA encapsulated in LNPs was also quantified by Quant-iT RiboGreen RNA assay (Life Technologies, Burlington, ON, Canada). Briefly, LNP-siRNA was incubated at 37 °C for 10 min in the presence or absence of 1% Triton X-100 followed by the addition of the RiboGreen reagent. The fluorescence intensity (Ex/Em: 480/520 nm) was determined using an absorbance and fluorescence dual spectrometer (Duetta, Horiba).

## Results

### Stability of carrier formulations treated at 37 °C

**Figure 1:**
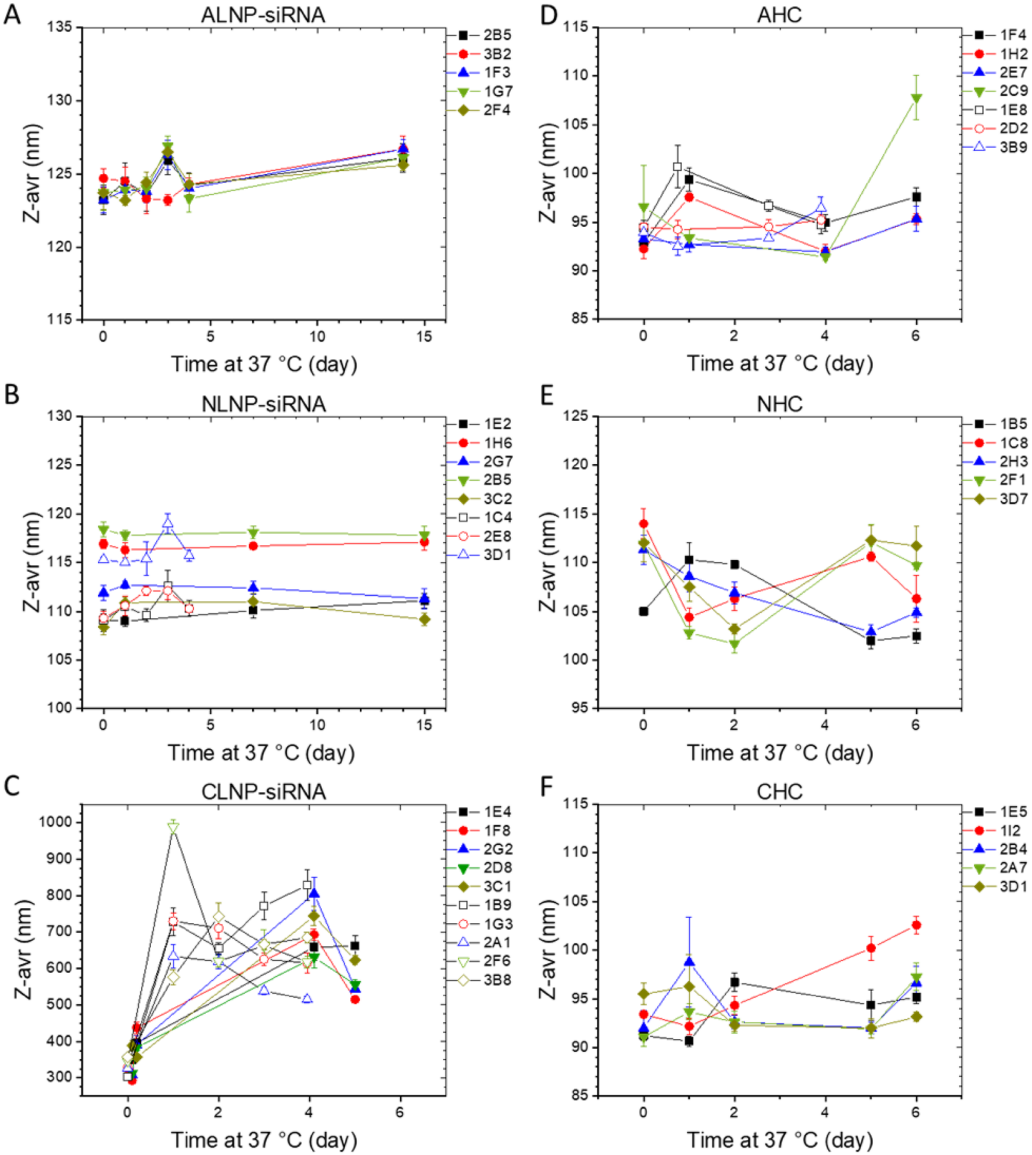
Size stability of anionic (**A,D**), neutral (**B,E**), and cationic (**C,F**) lipid nanoparticles (**A–C**), and liposomes (**D–F**). Each point is a Z-avr value obtained from five indications with the corresponding standard deviation depicted by the error bars. Solid lines connect points corresponding to the same vial and are shown to guide the viewer’s eye.

LNP-siRNA and liposome (HC) formulations were produced by INT and stored at -70 °C. First, LNP-siRNA and HC formulations stored (at − 70 °C) for less than 2 months were thawed at RT, and the Z-avr and PdI were measured (10 vials each; Table 2, denoted as “a”). In order to assess effects of the elevated incubation temperature on the particle size and size dispersion, Z-avr and PdI measurements were carried out with 5–8 vials of each formulation, first thawed at RT and then incubated at 37 °C, over the period of several days (Table 2). As evident from Fig. 1 and Table 2, despite visible day-to-day variation, no statistically significant change of the mean Z-avr size was observed for the three liposome (Fig. 1D–F) and two of the three lipid nanoparticle (ALNP and NLNP) formulations even after incubation at 37 °C for over two weeks (Fig. 1A,B). In contrast, an increase Z-avr size and somewhat smaller increase of PdI values was observed for CLNP between day 0 and day 1 with both measurands remaining statistically unchanged in the following days. Thorough examination of the CLNP measurement results for days 0 to 5 indicated a significant increase of the correlation function values at long correlation times from day 1 on. While effective Z-avr values could still be determined from a cumulants fit over a limited correlation function range with values greater than approximately 0.25, the presence of an aggregated or agglomerated fraction rendered the cumulants analysis unreliable. A follow up analysis of the particle size distribution by NNLS method (Zetasizer Software, ver. 8.0 research) showed in day 0 measurements a dominant fraction of the primary particles. However, a small tail of larger particles consistent with an onset of aggregation was also present. It should be emphasized though that no further particle size increase of formulation destabilization was observed for the 37 °C incubation of CLNP between day 1 and day 5 (Fig. 1C). A similar mean particle size increase could have been present in the toxicity samples, and therefore, a thorough systematic investigation of the effects of the particle size and formulation stability of LNP-siRNA biocompatibility will be required.

**Table 2:** Z-average and PdI of three LNP-siRNA and three liposome formulations, Z-avr and PdI values shown are the average values of n samples measured on a particular day and SD values are the corresponding standard deviations, respectively.

Formulations	Z-avr ± SD (nm)			
	0d	1d	2d	4d (5d*)
Anionic LNP-siRNA (2 mg/mL, n = 5)	123.7 ± 0.6	124.0 ± 0.5	123.8 ± 0.4	124.0 ± 0.4
	127.3 ± 0.5^a^			
Neutral LNP-siRNA (3 mg/mL, n = 8)	112.3 ± 4.0	112.9 ± 3.2	112.4 ± 2.9	112.1 ± 3.1
	85.8 ± 0.2^a^			
Cationic LNP-siRNA (2 mg/mL, n = 5)	332 ± 21	731 ± 158	670 ± 56	652 ± 116
	245.8 ± 0.9^a^			
Anionic Liposome (2 mg/mL, n = 7)	94.0 ± 1.4	95.8 ± 3.4	94.9 ± 1.7	93.8 ± 2.0
	90.5 ± 0.2^a^			
Neutral Liposome (2 mg/mL, n = 5)	110.8 ± 3.4	106.7 ± 3.1	105.6 ± 3.2	108.0 ± 5.1*
	105.8 ± 0.3^a^			
Cationic Liposome (2 mg/mL, n = 5)	92.6 ± 1.8	94.3 ± 3.2	93.7 ± 1.9	103.0 ± 14.3*
	90.7 ± 0.3^a^			

Formulations	PdI ± SD			
	0d	1d	2d	4d (5d*)
Anionic LNP-siRNA (2 mg/mL, n = 5)	0.047 ± 0.008	0.056 ± 0.007	0.046 ± 0.005	0.052 ± 0.006
	0.031^a^			
Neutral LNP-siRNA (3 mg/mL, n = 8)	0.182 ± 0.009	0.173 ± 0.013	0.191 ± 0.008	0.169 ± 0.012
	0.119^a^			
Cationic LNP-siRNA (2 mg/mL, n = 5)	0.266 ± 0.041	0.426 ± 0.134	0.400 ± 0.041	0.344 ± 0.111
	0.061^a^			
Anionic Liposome (2 mg/mL, n = 7)	0.108 ± 0.018	0.118 ± 0.035	0.108 ± 0.027	0.105 ± 0.027
	0.059^a^			
Neutral Liposome (2 mg/mL, n = 5)	0.136 ± 0.030	0.151 ± 0.034	0.132 ± 0.036	0.159 ± 0.051*
	0.092^a^			
Cationic Liposome (2 mg/mL, n = 5)	0.119 ± 0.030	0.137 ± 0.043	0.138 ± 0.024	0.169 ± 0.076*
	0.097^a^			

^a^Measured values at < 2 months storage (n > 10): the weighted average for PdI and standard deviations of repeatability for Z-avr were also shown.

*Indicates the values of 5d measurements.

### Cytotoxic effect of LNP-siRNA with high doses in adherent and non-adherent cell lines

**Figure 2:**
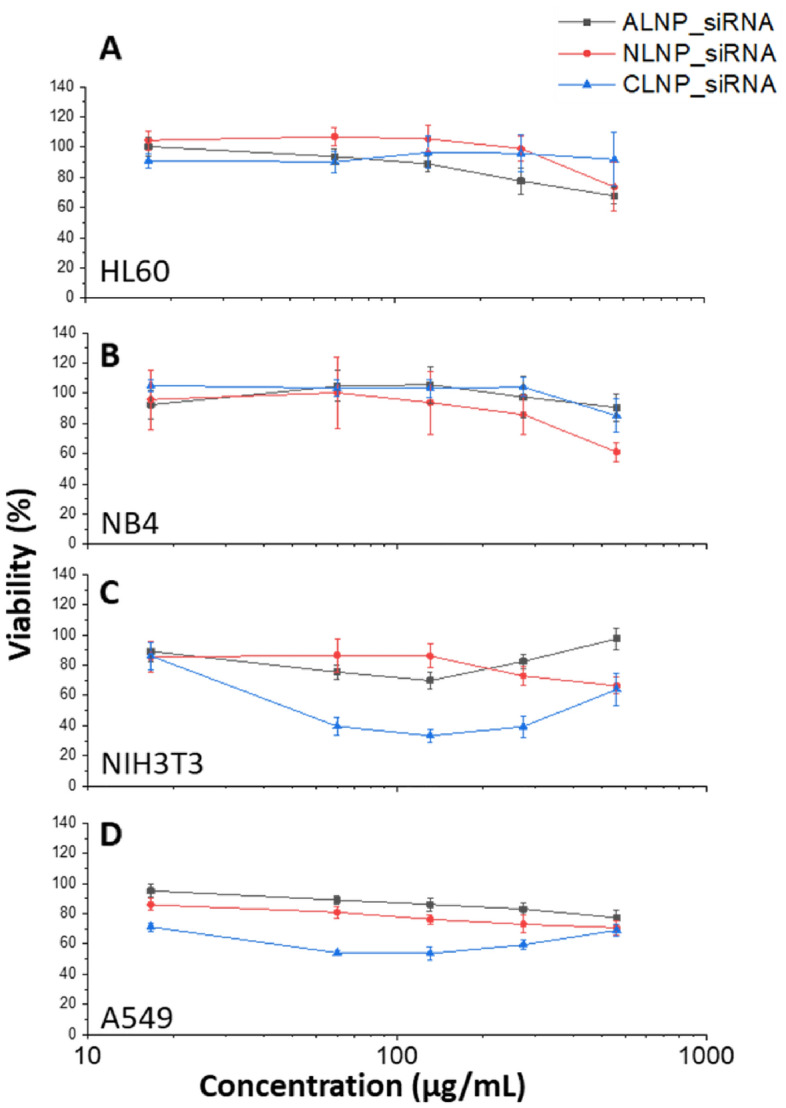
Toxicity of high concentration of lipid nanoparticles (LNP-siRNA) in adherent and non-adherent cells. Line graphs show the viability (%) after the treatment with each type of LNP-siRNA in non-adherent (**A**) HL60, (**B**) NB4 and adherent (**C**) NIH3T3, and (**D**) A549 cells at 24 h. Measurements were carried out in 6 wells for each concentration and each line graph represents the mean viability (%) observed with 3 vials of each LNP-siRNA.

It is generally believed that lipid-based nanoparticles display low or no toxicity particularly with lower concentration range of treatment^29,30^. However, high concentration of LNPs is required for a stable and long term storage. Therefore, high concentration of up to 0.5 mg/mL was employed for the cell proliferation assay tests in adherent and non-adherent cell lines. A wide range of concentration (0–512 µg/mL) of each LNP-siRNA formulation was tested by using three randomly drawn vials of each formulation in four cell lines. Cytotoxic effect of LNP-siRNA was measured on adherent (A549 and NIH3T3) and non-adherent (HL60 and NB4) cell lines. The line graphs (Fig. 2) for each LNP-siRNA represent the mean viability of the 3 vials tested for each formulation. The highest concentration used here was approximately 20–200 times higher than the doses commonly used in the literature to investigate gene silencing or expression in mammalian cells using LNP-siRNA or LNP-mRNA, respectively^31–33^.

There was no significant toxic effect of all three LNP-siRNA formulations up to 256 µg/mL (much higher than usual concentration in gene expression studies) in HL60 and NB4 cells (Fig. 2A,B). HL60 cells treated with 512 µg/mL of ALNP, NLNP and CLNP, respectively showed cell viability of 68, 74 and 92%. Similarly, another suspension cell line, NB4 exhibited percentage viability of 91, 61 and 85% when they were treated with ALNP, NLNP and CLNP, respectively. However, treatment of adherent cells (NIH3T3 and A549) with CLNP showed considerable toxicity starting at 128 µg/mL (viability observed was 33 and 54%, respectively, Fig. 2C,D). This concentration of CLNP is much higher than the routine ones. ALNP and NLNP in A549 cells at 512 µg/mL did not reduce the cell viability (71 and 77%, respectively).

### Systematic toxicity assessment of LNPs-siRNA and liposomes in HL60 cells and A549 cells

**Figure 3:**
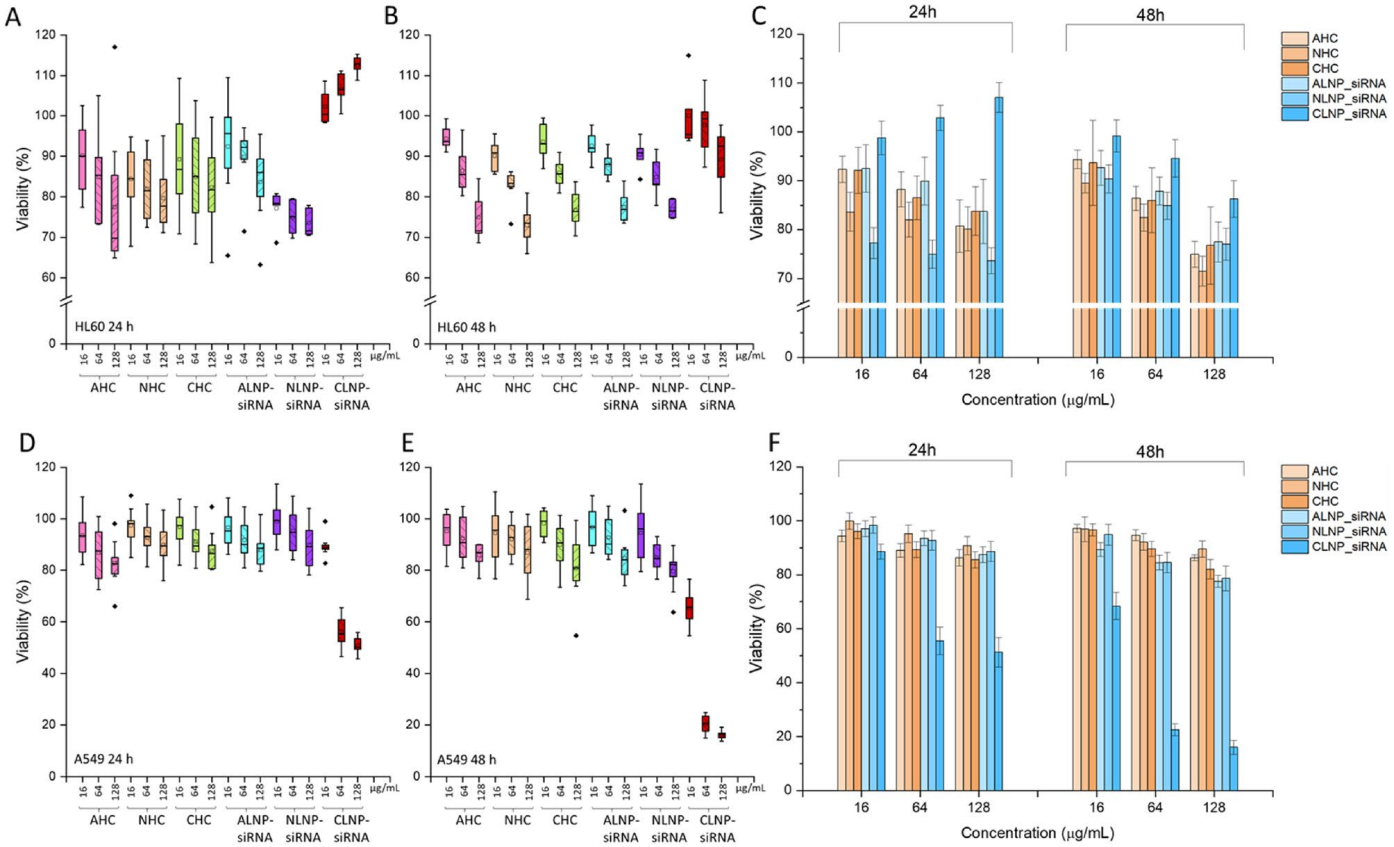
Toxicity of lipid nanoparticles (LNP-siRNA) and liposomes in HL60 (**A–C**) and A549 (**D–F**) cells. Box plots (**A,B,D,E**) show the range of cell viability (%) when cells were treated with 8–10 vials of each formulation. Bar graphs (**C,F**) summarize the average cell viability after the treatment with each type of LNP-siRNA and liposomes for 24 h and 48 h. Measurements were carried out in 6 wells for each concentration. Each bar represents the mean viability (%) observed in two rounds of measurements with 8–10 vials of each formulations. Note: Same vials were tested in both rounds.

In view of the somewhat toxic behavior of LNP-siRNA observed when cells were treated with more than 200 μg/mL, we systematically evaluated toxicity with all six formulations of liposomes and LNP-siRNA in acute myeloid leukemia cell line, HL60. In culture, these cells remain in suspension and are more sensitive to any external factors as compared to adherent epithelial cells. Moreover, differentiated HL60 cells were previously reported to be an easily accessible and suitable in vitro test model for assessing the cytotoxic effect of solid lipid nanoparticles^34^. The cytotoxic effect of the six formulations was evaluated by the WST-8 cell proliferation assay. HL60 cells were treated with varying concentrations (16, 64 and 128 µg/mL) of LNPs and HCs for 24 and 48 h. Ten vials each of the three types of liposomes and ALNP, and eight vials of NLNP and CLNP were tested for the cytotoxic effect. The experiments were repeated in duplicate rounds and measurements in six wells were carried out in each round (Fig. 3A,B). Overall, there was no significant toxicity observed with any of the LNPs or liposomes even at 48 h treatment and high concentration (Fig. 3A–C). At 24 h, percentage cell viability was ≥ 82, ≥ 77, and ≥ 112% in cells treated with 128 µg/mL ALNP, NLNP and CLNP, respectively. HL60 cells showed viability of ≥ 77, ≥ 80 and ≥ 89%, respectively, when treated with 128 µg/mL ALNP, NLNP and CLNP at 48 h. Liposomes, AHC, NHC and CHC treatment for 48 h at 128 µg/mL resulted in cell viability (%) of ≥ 75, ≥ 73 and ≥ 77%, respectively.

LNP-siRNA and liposomes were also tested for their cytotoxic effect on A549 cells. ALNP, NLNP, AHC, NHC and CHC were non-toxic to A549 cells at 24 and 48 h at all concentrations tested, CLNP did not affect cell viability (> 75%) up to 16 µg/mL at 24 and 48 h. Viability dropped down to below 25% when cells were treated with CLNP at 64 and 128 µg/mL at 48 h (Fig. 3D–F). These higher concentrations are generally not used for gene expression/silencing studies. The cell proliferation assay on 3D culture of A549 and HL60 cells with cationic LNP-siRNA did not impact their viability (~ 80%) after 48 h, even at high concentration (Figure S1).

### Effect of extended treatment of LNP-siRNA in adherent and non-adherent cells

**Figure 4:**
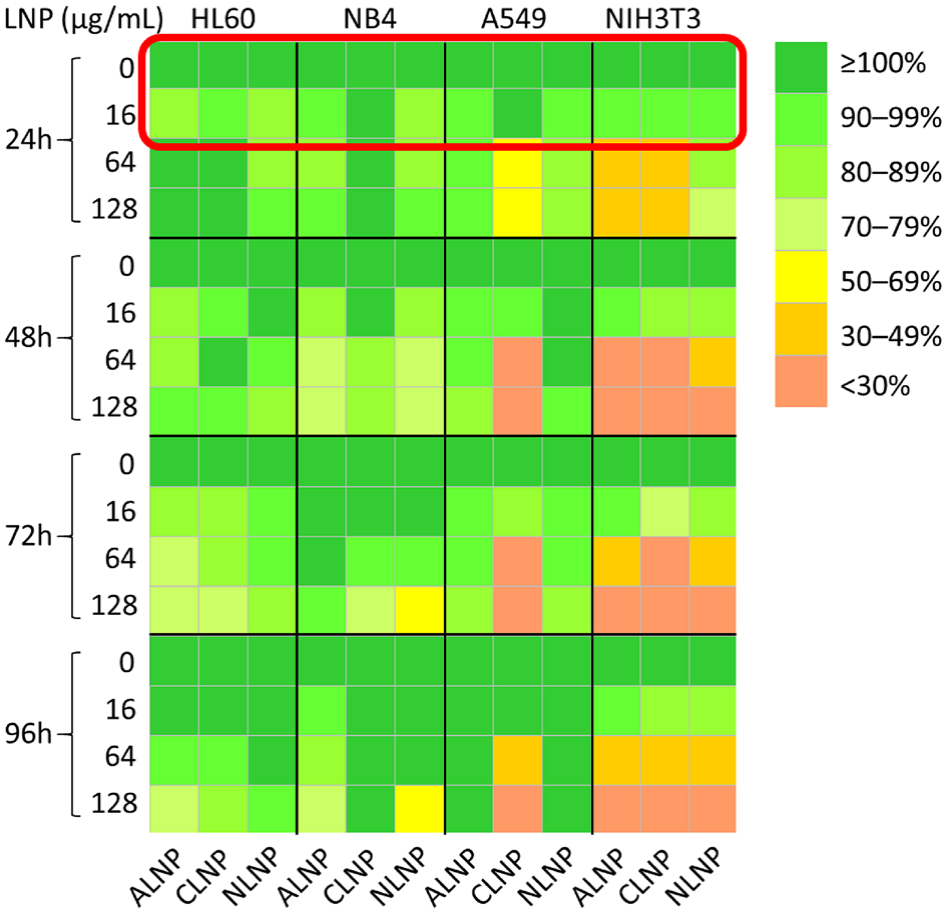
Effect of longer incubation time of LNP-siRNA in adherent and non-adherent cells (the labels are in abbreviation form, LNP: LNP-siRNA). Contour map shows the mean viability (%) after the treatment with each type of LNP-siRNA in HL60, NB4, A549 and NIH3T3 cells up to 96 h. Measurements were carried out in 6 wells for each concentration and each cell represents the mean viability (%) calculated from the 6 wells. Red color rectangle denotes the range of conventionally used concentration in gene silencing/expression studies.

In order to assess if increased duration of LNP-siRNA treatment has any toxic effect on cell lines, WST assay was performed in HL60, NB4, A549 and NIH3T3 cells, after 24, 48, 72 and 96 h with each of the LNP-siRNA. None of the LNP-siRNA formulations caused the viability (%) of suspension cells to below 50% (Fig. 4) except 128 µg/mL of NLNP in NB4 cells. All three types of LNP-siRNAs were non-toxic to both adherent cell lines, A549 and NIH3T3 up to 16 µg/mL at all time points tested. However, cationic LNP-siRNA > 64 µg/mL was toxic to A549 cells after 48 h, while NLNP and ALNP had little impact on cell viability. Interestingly, NIH3T3 cells appeared to be more sensitive to all three types of LNP-siRNA than A549, and showed reduced viability at > 64 µg/mL.

### Quantification of LNP-siRNA

The amount of siRNA present in each formulation of LNP-siRNA was quantified by absorbance and RiboGreen fluorescence assays (Table 3). First, a standard siRNA calibration curve was determined in the presence of each lipid film formulation. Next, dilutions of LNP-siRNA formulations were prepared in a 1%SDS-0.5X dPBS buffer and absorbance spectra were measured. Finally, the concentration of siRNA in each formulation was calculated from the respective standard siRNA calibration curves (Table 3). UV absorbance spectra for various reference solutions with all lipids not in LNP form were also recorded (Figure S2). Absorbance at 260 nm for each reference solution was below 0.02 except for lipid in sucrose-1% SDS-0.5X dPBS which was around 0.13. Sucrose is included in the LNP-siRNA formulations as a cryoprotectant. Lipids at high concentration showed absorbance close to or higher than 0.1 at 260 nm, which indicated a possible interference with the LNP-siRNA absorbance measurements. Though we included the reference solution to have the respective lipids in 0.5X dPBS containing sucrose and SDS, from our observation, it is concluded that not all of the compositions in the LNP-siRNA have been taken into account.

**Table 3:** Concentrations (in µg/mL) of nucleic acid (readings from the NanoDrop based on the absorbance assay) and siRNA determined by absorbance and RiboGreen fluorescence assay.

	Nucleic acid (A260/A230)	siRNA (absorbance)	siRNA (RiboGreen assay)
ALNP	87 ± 2	96 ± 2	112 ± 11
NLNP	90 ± 3	106 ± 2	217 ± 17
CLNP	78 ± 2	87 ± 2	109 ± 8

RiboGreen assay allows the detection of small quantity of nucleic acids, with linear fluorescence detection for RNA in the range of 1–200 ng. The concentration of siRNA in the LNP-siRNA formulations was determined and the results were slightly higher than the ones obtained from the absorbance measurements for ALNP (112 vs. 96 µg/mL) and CLNP (109 vs. 87 µg/mL), but approximately twice as large (217 vs. 106 µg/mL) for the NLNP-siRNA (Table 3).

## Discussion

This report demonstrates the (non-)cytotoxic profile of the six candidate lipid based formulations developed by INT, in order to establish certified reference materials for liposomes and LNP-siRNAs. Three formulations of liposomes evaluated in this report did not affect the proliferation of both adherent and non-adherent cells. All three formulations of LNP-siRNAs were also non-toxic in suspension cells at all concentrations tested and did not affect the viability of adherent cells at typically used concentration up to 16 µg/mL.

Systematic evaluation of three liposomes and three LNP-siRNAs, each of 10 vials with duplicate samples showed that more than 75% of HL60 cells were viable even when treated with 128 µg/mL of LNP-siRNA for 48 h. Similarly, other reports also demonstrated non-toxic effect (> 90% cell viability after 48 and 72 h of incubation) of solid lipid nanoparticles in HEK293 cells^35,36^. Solid lipid nanoparticles showed low toxicity in HL60 cells^34^ as well. Moreover, liposomes, anionic and neutral LNP-siRNAs were non-toxic to A549 cells even at 128 µg/mL. Cationic LNP-siRNA did not affect cell proliferation of A549 cells up to 16 µg/mL and exhibited cytotoxicity only above 64 µg/mL, which is typically not used for gene expression or silencing studies in vitro. Usually, lipid formulations are used in the range of 2–25 µg/mL for gene expression/silencing studies^31–33^.

The Z-avr value for 10 vials of CLNP at 37 °C ranged from 300 to 800 nm, which is relevant in the toxicity measurements. The significant change in their Z-avr values of CLNP may have an impact on their cytotoxic behavior at higher concentration (≥ 64 µg/mL). These measurements suggest the fact that the stability of the lipid formulations may influence their biocompatibility and also the potency of these carriers. A thorough and systematic investigation is required to understand the relation between size and PdI measurements with the efficacy of these lipid based nanoparticles.

Although it was previously reported that cationic liposomes cause hemolysis and hepatotoxicity due to their electrostatic interaction with negatively charged cellular components and fusion with plasma membrane^19,37,38^, our study showed that cationic LNP-siRNA were non-toxic up to 16 µg/mL in adherent cells. Moreover, all three types of LNP-siRNAs exhibited low cytotoxicity to both suspension cell lines up to 256 µg/mL, which is 20–200 times higher than typically used concentration in gene expression/silencing studies^31–33^. Extreme concentration (such as 512 µg/mL) of LNP-siRNAs were tested in order to understand the effect of very high concentration and also due to the in-house availability of these formulations in large volumes.

Anionic, neutral and cationic liposomes with siRNA were previously reported to be low cytotoxic with longer circulation time and exhibited efficient knockdown of the targeted protein^38^ in breast cancer cells. The lower toxicity effect of ALNP, NLNP and CLNP in this study supports this previous observation.

Three dimensional culture is much more relevant as they recapitulate the in vivo conditions. It was demonstrated earlier that proliferation and gene expression pattern of three-dimensional spheroids is similar to in vivo tumors^39^. The spatial organization in a 3D model affects the cellular functions like growth, cell–matrix and cell–cell interaction, drug resistance and all these arise from the group of cells and not from single cells^40^. Determination of toxicity or drug resistance in 3D culture models or other biomimetic models are much more pertinent and therefore, can be translated^41^. In this report, considerable toxicity was observed in A549 cells with CLNP only at 64 µg/mL and above. Therefore, we decided to evaluate the toxicity of cationic LNP-siRNA on cell proliferation of HL60 and A549 cells in 3D conditions. Low toxicity observed in 3D culture suggests that CLNP may be considered as drug carriers along with anionic and neutral LNP-siRNAs.

Extended treatment of LNP-siRNAs on suspension cells, HL60 and NB4, did not have an impact on their proliferation up to 64 µg/mL. In A549 cells, anionic and neutral LNP-siRNAs were non-toxic at all concentrations and cationic LNP-siRNA up to 16 µg/mL. Interestingly, high concentration (≥ 64 µg/mL) of all 3 formulations of LNP-siRNAs reduced the cell viability of NIH3T3 cells, likely due to their susceptibility to transfection as shown in the literature^42^. It is noteworthy that the highest concentration used in this work was approximately 5–50 times higher than typically used in gene silencing or expression experiments in cultured cells (2.5 to 25 µg/mL is typically used)^31–33^.

Quantification of LNP-siRNA by absorbance and fluorescence based RiboGreen assay showed comparable results for ALNP and CLNP, but was different for NLNP-siRNA. Absorbance at 260 nm for each reference solution was below 0.1 except for lipid in sucrose-1% SDS-0.5X dPBS (~ 0.13). Sucrose is included in the LNP-siRNA formulation to maintain the stability at lower temperatures. The higher concentration of lipid showed absorbance close to 0.1 at 260 nm and this seemed to be interfering with the actual measurements of LNP-siRNA. Though we took into account for the absorbance and scattering of reference solution including all lipid components in 0.5X dPBS containing sucrose and 1% SDS, from our observation, it is concluded that there are still missing components in the reference measurements. For example, the interactions of lipids with sucrose have not been considered which may not reflect the exact composition of the SDS treated LNP-siRNAs, further investigation will be needed to figure out the discrepancy between the measurements.

## Conclusions

All three formulations of liposomes and LNP-siRNAs were non-toxic to suspension cells even at higher concentrations. There was no effect of all 3 types of liposomes, anionic and neutral LNP-siRNAs on the proliferation of A549 cells. Cationic LNP-siRNA up to 16 µg/mL was non-toxic to adherent cells. Reduced viability of adherent cells below 50%, observed on treatment with CLNP (≥ 64 µg/mL) may be due to the significant variation in the size measurements at 37 °C. Further research is required to elucidate the exact formulation of reference solutions in order to quantify the siRNA in the lipid nanoparticles. This report demonstrates the cytotoxic profiles of the initial candidate CRM formulations. More candidates are being developed and will be evaluated for size, toxicity and efficacy in both adherent and non-adherent cells to establish a certified reference material.

## Supplementary Information

Supplementary Information.

## Data Availability

All data generated or analyzed during this study are included in this published article and its supplementary information files. The composition of the formulations is not publicly available due to the IP and treat secret policy at INT, but some details are available from the corresponding author on reasonable request.
