# Supplementary material for: Development of lipid nanoparticles and liposomes reference materials (II): cytotoxic profiles
Source: Sci Rep. 2022 Oct 27;12:18071. doi: 10.1038/s41598-022-23013-2 (PMC9610362; doi:10.1038/s41598-022-23013-2)
Supplement: Supplementary file 1 — Supplementary Information. [file 41598_2022_23013_MOESM1_ESM.docx]

Supplementary materials: Development of lipid nanoparticles and liposomes reference materials (II) - Cytotoxic profiles

Krishnapriya Syama ^1^, Zygmunt J. Jakubek ^1^, Sam Chen ^2^, Josh Zaifman ^2^, Yuen Yi C. Tam ^2^, Shan Zou ^1,*^

^1^ Metrology Research Centre, National Research Council Canada, Ottawa, Ontario, Canada

^2^ Integrated Nanotherapeutics, Burnaby, BC, V5G 4X4, Canada

^*^ Dr. Shan Zou, Metrology Research Centre, National Research Council, Canada, 100 Sussex Drive, Ottawa, Ontario K1A 0R6, Canada. Email: Shan.Zou@nrc-cnrc.gc.ca (ORCID: 0000-0002-2480-6821)

**Details of formulations**

The anionic, neutral, and cationic LNP formulations (ALNP, NLNP, CLNP, respectively) were produced by rapidly mixing the lipid components dissolved in ethanol with siRNA in aqueous buffer at a volumetric flow rate ratio of 1:3 (ethanol to aqueous, combined flow rate 28 mL/min) at room temperature. The product was then dialyzed against 1x Dulbecco's phosphate-buffered saline (dPBS) (Gibco, Thermo Fisher Scientific, Canada) at pH 7.4 for 24 hours to remove residual ethanol and to raise pH. The anionic, neutral, and cationic liposome formulations (AHC, NHC, and 77 CHC, respectively) were produced by forcing appropriate lipid mixtures in 0.5x dPBS and 15% sucrose 12−15 times under 300 PSI pressure through two-stacked 0.08 µm polycarbonate membranes using the Lipex liposome extruder heated to 65 ºC. The LNP and liposome formulations were diluted with dPBS and aqueous sucrose solution to achieve the final lipid concentration in 0.5 dPBS/15% sucrose equal to 2 mg/mL except for NLNP that was 3 mg/mL. siRNA loaded into lipid nanoparticles amounted approximately 4.0 wt% of the total mass for ALNP and NLNP and 3.3 wt% for CLNP. Sets of 200 sequentially numbered vials, 1 mL/vial, were produced for each formulation; limited number of extra vials were also retained at INT for future reference. Vials were frozen in a -70ºC freezer. The boxed sets of 200 vials each were shipped overnight to NRC in thermally insulated styrofoam containers on dry ice.

**Zeta potential measurements**

Zeta potential values were measured with Zetasizer Nano ZS by mixed mode measurement phase analysis light scattering. Aliquots of 100 µL formulations were diluted tenfold in water for injection (WFI) (HyClone, Fisher 155 Scientific, Canada) to obtain the target concentration of 300 µg/mL for NLNP and 200 µg/mL for the other formulations. All dilutions were prepared in a disposable 4 mL polystyrene cuvette and subsequently loaded into a folded capillary cuvette (DTS1070, Malvern Panalytical, UK) with a 1 mL syringe. Measurements were conducted at (25.0±0.1) ºC and 50 V applied voltage. Following minimum 3 minute-long sample equilibration in the sample compartment, five repeat indications separated by a 30 s pause were recorded for each measurement with minimum 10 and maximum 100 runs for each indication. The measurements were controlled and raw data processed and analyzed with the Zetasizer Software using default parameters except for those listed in Table 1. Finally, pH of undiluted (and thawed) formulations was monitored using an Accumet pH meter and a micro probe (Fisher Scientific, Canada).

**
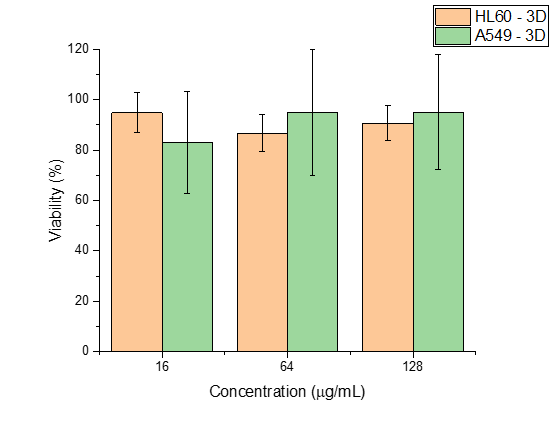
**

**Figure S1.** Cell viability (%) of cationic lipid nanoparticle (CLNP- siRNA) treated HL60 and A549 cells in 3D cultures for 48 h. Measurements were carried out in 6 wells for each concentration.

**
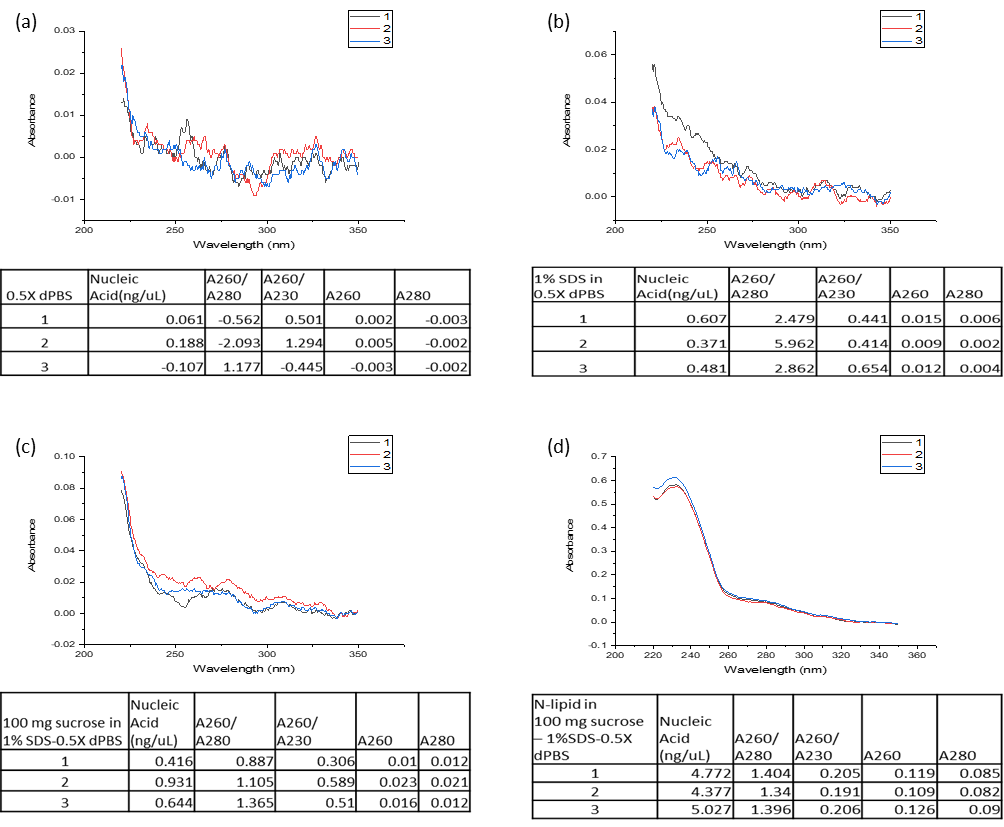
**

**Figure S2.** Absorbance measurements with reference solutions of (a) 0.5X dPBS, (b) 1% SDS in 0.5X dPBS, (c) 100 mg (2%) sucrose in 1% SDS-0.5X dPBS and (d) lipid film of neutral LNP formulations in 2% sucrose -1% SDS-0.5X dPBS. Three repeats were shown in each set.
